# Transcriptional Enhancers in the Regulation of T Cell Differentiation

**DOI:** 10.3389/fimmu.2015.00462

**Published:** 2015-09-09

**Authors:** Michelle L. T. Nguyen, Sarah A. Jones, Julia E. Prier, Brendan E. Russ

**Affiliations:** ^1^Department of Microbiology and Immunology, The Peter Doherty Institute for Infection and Immunity, The University of Melbourne, Melbourne, VIC, Australia; ^2^Monash University Centre for Inflammatory Disease, School of Clinical Sciences at Monash Health, Melbourne, VIC, Australia

**Keywords:** transcriptional enhancers, transcription factors, T cell differentiation, epigenetics, differentiation

## Abstract

The changes in phenotype and function that characterize the differentiation of naïve T cells to effector and memory states are underscored by large-scale, coordinated, and stable changes in gene expression. In turn, these changes are choreographed by the interplay between transcription factors and epigenetic regulators that act to restructure the genome, ultimately ensuring lineage-appropriate gene expression. Here, we focus on the mechanisms that control T cell differentiation, with a particular focus on the role of regulatory elements encoded within the genome, known as transcriptional enhancers (TEs). We discuss the central role of TEs in regulating T cell differentiation, both in health and disease.

## Introduction

The first T cell fate decision occurs in the thymus, where CD4^+^CD8^+^ (double positive) T cells selectively and stably down-regulate CD4 or CD8 before exiting the thymus as mature, single positive (CD4^+^ or CD8^+^), naïve T cells. This initial decision dictates the range of possible fate outcomes for each lineage of T cells, and hence the role that they play in host immunity; while being able to adopt at least six distinct differentiation states, including Th1, Th2, Th17, and Treg fates, each with specific phenotypic and functional characteristics, T cells entering the CD4^+^ T cell pathway contribute to pathogen clearance primarily through the secretion of cytokines that coordinate the activity of other immune cells [reviewed in Ref. (1)]. For instance, CD4^+^ T cells that adopt the Th2 phenotype secrete IL-4, which elicits maturation of the B cell-mediated antibody response while regulatory T cells (Tregs) function to maintain immune homeostasis through the secretion of immunosuppressive cytokines, including IL-10 and TGF-β. CD8^+^ T cells appear to have a much more limited range of fate choices, and their predominant function is in the direct killing of infected or transformed cells via the secretion of cytotoxic molecules, including perforin and the granzymes [reviewed in Ref. (2)]. Despite these differences in the range of lineage decisions that CD4^+^ and CD8^+^ T cells can make, pathogen-induced differentiation of T cells of either lineage follows the same general course; pathogen recognition results in the differentiation of naïve, quiescent T cells to short-lived effector cells that have gained the capacity to express the effector molecules required to clear the infection. Importantly, differentiation of naïve T cells results in the generation of long-lived memory cells, which relative to naïve cells, respond more readily and rapidly to subsequent infections with the same pathogen, providing the basis of T cell-mediated immunity (3, 4). It is currently unclear as to whether memory cells differentiate directly from naïve cells, or must first become effector cells. While it is understood that the phenotypic and functional differences between cell types are underscored by cell type-specific transcriptional profiles (5, 6), how these unique profiles arise and are maintained is far from resolved. Here, we focus on the mechanisms that control the establishment and maintenance of T cell identity, with a particular focus on the role of genomic elements known as transcriptional enhancers (TEs). TEs are DNA sequences often occurring tens to hundreds of kilobases away from their cognate gene, which activate gene transcription by recruitment of transcription factors (TFs) to the gene locus. While TEs were discovered more than 30 years ago (7), they have recently become the focus of renewed interest as evidence accumulates that they play central roles in cell fate decisions, and as advances in technology enable them to be studied more readily.

## Molecular Control of Cellular Differentiation

Cellular differentiation results from specific and stable changes in gene transcription, such that lineage-appropriate genes are heritably up-regulated, while non-lineage genes are silenced. Thus differentiation requires both a mechanism to initiate specific transcriptional changes, and another to reinforce those changes, allowing stability of the new transcriptional program, within and between cell divisions. These aspects of differentiation are linked by the interplay between TFs and chromatin modifying proteins, where specificity relies on the precise binding of TFs to specific sequence motifs occurring within gene regulatory regions (gene promoters and enhancers). Transcriptional changes are then enabled by transcription factor-mediated recruitment of chromatin, modifying proteins to specific regions of the genome. Following recruitment, chromatin modifiers catalyze localized and often heritable changes to the genome structure, making the DNA template more or less permissive for transcription, thus, resulting in gene activation or silencing (8). Since the TFs that drive T cell differentiation have been the focus of a number of thorough and recent reviews (9–12), this article will focus specifically on regulated changes to the chromatin structure that guide T cell fate, with a particular focus on TEs.

## Control of Gene Expression by Modulation of the Chromatin Structure

Within eukaryotic cells, the negatively charged DNA is wound around positively charged histone protein complexes called nucleosomes. This arrangement, termed chromatin, enables meters of DNA to fit into a nucleus with a diameter measured in microns, but also provides an obstacle to transcription since compaction of the genome can occlude access of the transcriptional machinery to the genes. As such the positioning of nucleosomes along the DNA strand must be tightly regulated to ensure that lineage-appropriate genes are accessible for transcription. Conversely, by regulated compaction of the DNA, inappropriate gene expression can be silenced. Indeed, modulation of localized genome compaction – achieved through regulated nucleosome positioning – is an important means by which cellular fate decisions are controlled (13, 14).

**Figure 1:**
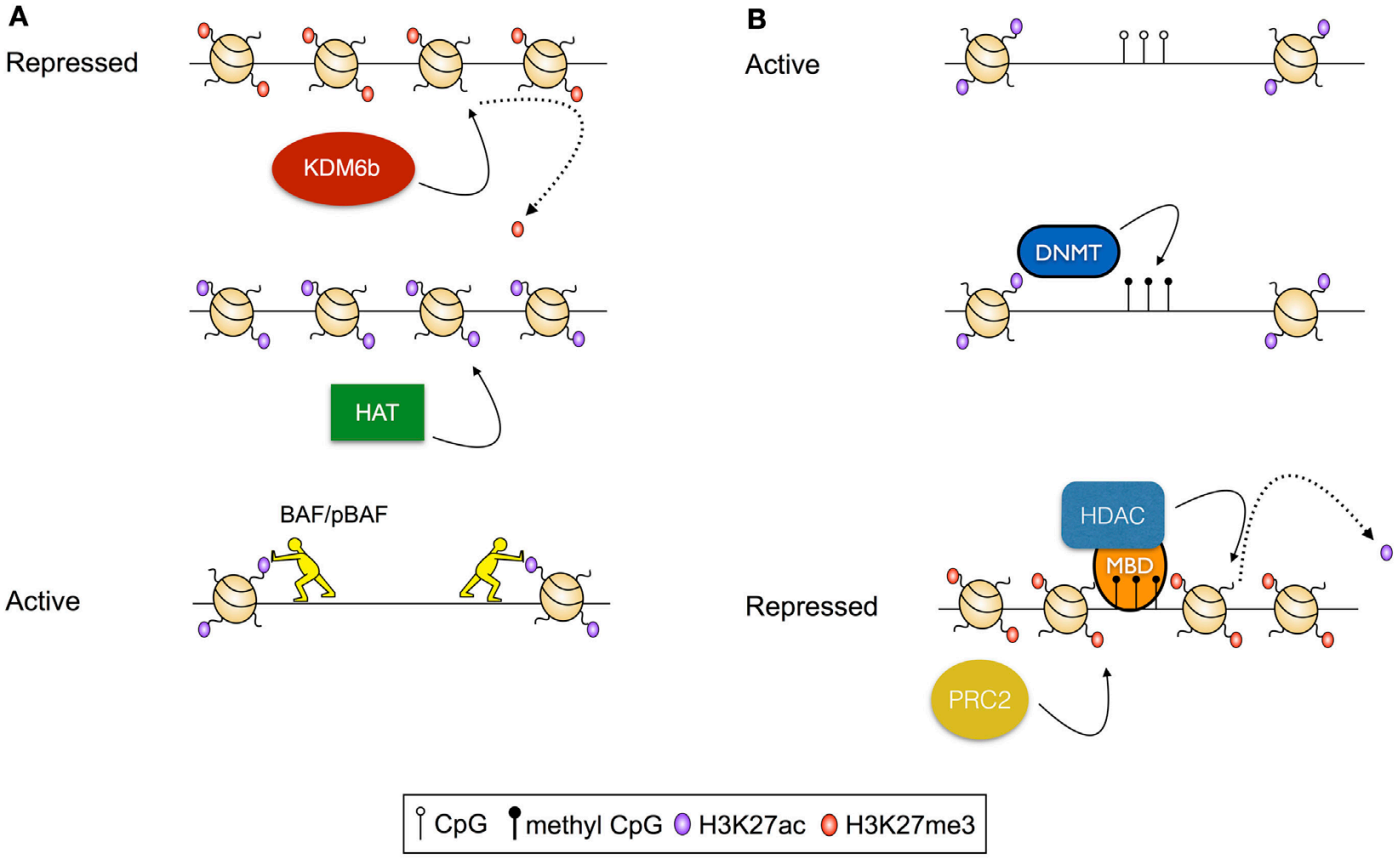
**Examples of transcriptional regulation via modulation of histone modifications**. **(A)** Activation of a repressed chromatin state: repressed chromatin is typified by deposition of histone modifications, including H3K27me3 and densely structured chromatin. In this scenario, activation results from demethylation of H3K27, modulated by the histone demthylase KDM6b, followed by acetylation of residues including H3K27 by a histone acetyl transferase (HAT). Finally, acetylated histones serve as substrates for histone remodeling complexes including BAF and pBAF, which reduce chromatin density, exposing the intervening DNA for access by transcription factors and the transcriptional machinery. **(B)** Repression of an active chromatin state: active chromatin is typified by acetylated histones, unmethylated CpG residues, and high chromatin accessibility. In this scenario, CpG methylation is catalyzed by a DNA Methyltransferase (DNMT). Methylated CpG residues then serve as a substrate for methyl-binding domain proteins (MBDs), which recruit histone deacetylases (HDACs). Following deacetylation, H3K27me3 is deposited by the Polycomb Repressive Complex 2 (PRC2).

The location of nucleosomes is regulated by modulated deposition and removal of post-translation modifications (PTMs) to the solvent-exposed N-termini of histones. These modifications can themselves direct the positioning of nucleosomes by altering the affinity of the histones for the DNA, or may serve as substrates for chromatin remodeling complexes that deposit, evict, and reposition nucleosomes. For instance, histone acetylation, which reduces the net positive charge on the nucleosome, and therefore the intimacy of the association between the DNA and the nucleosome, is thought to destabilize dense chromatin structures, making localized regions of the genome more accessible for transcription (15). Further, acetylation of sequential nucleosomes – a characteristic of regulatory regions of actively transcribed genes – results in charge repulsion, exposing the intervening DNA for access by the transcriptional machinery. Acetylated histones can also serve as substrates for the binding of chromatin remodeling complexes, including the ATP-dependent BAF (BRG1- or HRBM-associated factors) and pBAF (polybromo-associated BAF) complexes (16, 17), which rearrange chromatin to increase DNA accessibility [reviewed in Ref. (18); Figure 1A]. In contrast, histone methylation is associated with both active and repressed chromatin states, depending on the precise residue modified, and the extent of modification (19). For example, trimethylation of lysine 27 of histone 3 (H3K27me3), deposited by the Polycomb Repressive Complex 2 [PRC2 (20)] and removed by histone demethylases KDM6b and UTX (21), is associated with repressed gene transcription. Alternatively, H3K4me3, deposited by the Trithorax group proteins and removed by the Jarid1 histone demethylases, is associated with active transcription [reviewed in Ref. (22)]. Furthermore, histone methylation apparently conveys effects on transcription solely through indirect means, acting as a substrate for nucleosome remodeling complexes. As an example, H3K4me3, which is commonly deposited at active gene promoters, serves as a substrate for binding of the chromodomain-containing remodeler CHD1, which appears to establish accessible chromatin structures by nucleosome repositioning (21).

Highlighting the role of modulated deposition of histone modifications as a factor controlling cellular differentiation, Wei et al. showed that the deposition of H3K27me3 and H3K4me3 at gene promoters was predictive of gene expression patterns within naïve and *in vitro* differentiated effector CD4^+^ T cells of several specifications (including Th1, Th2, and Treg phenotypes) (23). Furthermore, they showed that “bivalent” gene promoters (baring both H3K27me3 and H3K4me3) within naïve cells often encoded TFs known to be the key for the establishment of effector T cell fates, including TBET and GATA3. These bivalent domains mostly resolved to either a transcriptionally repressive (H3K27me3^+^ H3K4me3^−^) or a transcriptionally permissive (H3K27me3^−^ H3K4me3^+^) state following effector differentiation, with the precise patterns largely reflecting the particular fate decision. For instance, Th1 differentiation was associated with the resolution of the *Tbx21* locus (encoding the Th1 defining TF TBET) to a permissive (H3K4me3^+^ H3K27^−^) signature, with a largely repressive (H3K4me3^+^ H3K27^−^) signature observed at the same locus in Th2 cells. Thus, it appears that bivalent domains represent switches that regulate fate specification.

Indicating that the mechanisms observed by Wei et al. also guide regulation of gene expression and differentiation in CD8^+^ T cells, and indeed are relevant *in vivo*, we have recently showed that changes in the transcriptional profiles of CD8^+^ T cells differentiating in response to an acute viral infection also correlated with changes in the deposition of H3K4me3 and H3K27me3, across the stages of differentiation, including memory (6). Moreover, we provided evidence for the coordinated regulation of functionally related genes via a mechanism dependent on modulated deposition of histone modifications, by showing that each group of genes had similar histone modification profiles in naïve cells, and similar patterns of change following differentiation. For instance, as per the Wei et al. study described above, bivalent gene promoters largely encoded TFs known to be the key for the establishment of effector and memory T cell fates, including TBET and EOMES (24). However, following differentiation, the repressive modification was selectively removed from the vast majority of these genes, coincident with their expression. Thus, it appeared that coordinated removal of the repressive modification from bivalent loci is a key step in activating genes required to establish T cell effector and memory fates. Moreover, similar coordination was evident for immune-related genes, such as interferon-γ (*Ifng*), which were characterized by gene promoters that were H3K27me3^+^ H3K4me3^−^ in naïve cells, but H3K27me3^−^ H3K4me3^+^ in effector and memory cells. Again, these changes coincided with gene activation following differentiation, highlighting the role played by histone PTMs in the regulation of cellular differentiation, and further suggesting that genes with similar functions are coordinately regulated via a mechanism dependent on regulated deposition of histone PTMs.

Aside from changes to the structure of the genome that are modulated via nucleosome remodeling, the structure of the DNA template can by modified by the addition and removal of methyl groups to the nucleotides of the genome. While multiple methylation states exist, the best characterized of these is 5-methyl cytosine, which occurs predominantly in the context of CpG dinucleotides, and is strongly associated with transcriptional repression (25). CpG methylation conveys its effects via stearic hindrance of protein–DNA interactions that would otherwise activate transcription (26, 27). Alternatively, methylated CpG residues act as substrates for methyl-CpG-binding domain proteins that, in turn, recruit histone deacetylases [HDACs (28–30)], and thus there is an interplay between CpG methylation-mediated mechanisms of transcriptional control and mechanisms dependent on histone post-translational modifications (Figure 1B).

## Enhancer Form and Function

Transcriptional enhancers are DNA sequences located within non-coding regions of the genome that positively regulate gene transcription through differentiation state-specific binding of activating TFs. In turn, TF binding catalyzes events that lead to the establishment of transcriptionally permissive chromatin environments, achieved through remodeling of the chromatin structure (described below). While the majority of studies of differentiation-dependent transcription have focused on gene promoters, it is clear that many of the regulatory events controlling cell fate decisions occur at TEs. Illustrating this point, a hallmark study by Heintzman et al. used genome-wide histone modification profiling in five human cell lines derived from various tissues, finding that the chromatin state of gene promoters was largely invariant across cell types. In contrast, the chromatin profiles of TEs were highly dynamic, and described much more closely the variation in cell type-specific gene expression than did the dynamics of gene promoters (31). Further, these findings have now been extended to many different primary cell types [(32, 33) reviewed in Ref. (34)].

Transcriptional enhancers were classically defined as short sequences of DNA that activate or increase transcription from gene promoters when studied in isolation, using plasmid-based reporter assays. These assays are typically performed using heterologous (often viral) promoters, demonstrating that TEs lack inherent promoter specificity. Moreover, enhanced transcription is observed regardless of the orientation in which the enhancer is cloned, and irrespective of the distance between the enhancer and promoter, reflecting the fact that, *in vivo*, TEs can occur hundreds of kilobases up or downstream of the genes that they regulate. Finally, mutational analysis shows that TE activity is dependent on the binding of TFs to motifs within the enhancer (35–37).

**Figure 2:**
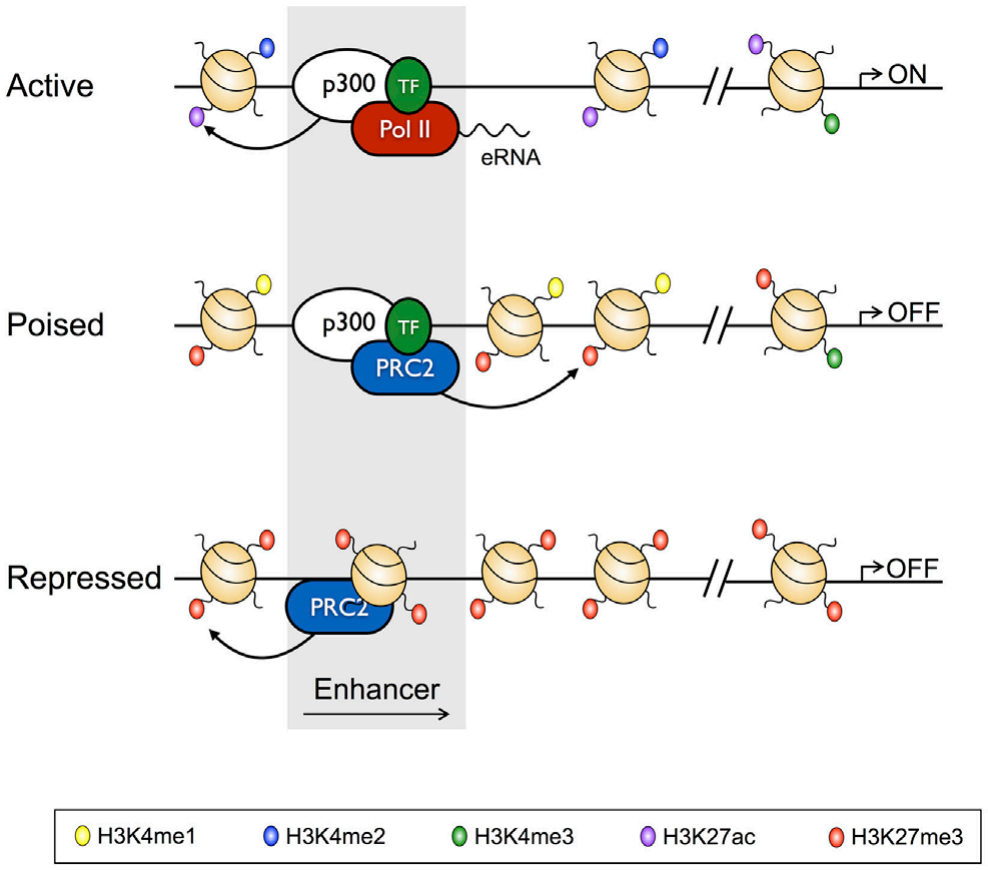
**Characteristics of active, poised, and repressed transcriptional enhancers and their cognate gene promoters**. Active transcriptional enhancers are bordered by widely spaced nucleosomes, baring modifications including H3K4me2 and H3K27Ac, and are bound by the histone acetyltransferase p300 and lineage-specific transcription factors. Active enhancers give rise to eRNAs, and are associated with promoters baring H3K27Ac and H3K4me3. Relative to active enhancers, poised enhancers do not give rise to eRNAs, have H3K27me3 in place of H3K27Ac, and have reduced chromatin accessibility. Poised enhancers are also bound by the Polycomb Repressive Complex 2 (PRC2), and their associated enhancers often have a bivalent signature. Finally, repressed enhancers are characterized by dense nucleosome assemblages baring H3K27me3, and are bound by PRC2.

With the recent advent of tools, such as ChIP-Seq, which have allowed the genome-wide distribution of proteins that interact with the genome to be studied, the classic definition of TEs has been further refined to show that, *in vivo*, TEs exist in poised, active, and repressed states, with each state characterized by unique histone PTM profiles, and unique TF and transcriptional co-regulator-binding profiles [reviewed in Ref. (38); Figure 2]. For instance, TEs of active genes are characterized by deposition of the activating H3K4me2 and H3K27Ac PTMs, while being depleted of the repressive H3K27me3 modification, and having low nucleosome density. On the other hand, repressed enhancers are enriched for H3K27me3, and depleted of activating modifications, and are nucleosome dense (14, 39). Poised enhancers represent a state between activation and repression, being defined by H3K4me1 and H3K27me3 deposition, increased chromatin accessibility relative to repressed enhancers, and binding by the p300 histone acetyltransferase, which also identifies active enhancers. However, unlike active enhancers, poised enhancers lack acetylated histones (40–42). Enhancers marked by this poised signature are not transcribed, but can be rapidly activated following differentiation, and as such, are reminiscent of bivalent domains (H3K4me3^+^ H3K27me3^+^) at gene promoters (6, 43). Moreover, like the genes marked by bivalent promoters, poised enhancers appear to regulate genes that enable cell fate specification (42). Thus, poised enhancers may represent “differentiation switches”, whereby their activation drives lineage commitment, while their repression blocks fates associated with the particular genes that they regulate. Finally, as with active enhancers, poised enhancers are bound by lineage defining TFs, which appear to be required to establish the cell state-specific “enhancersomes” [(44) described further below].

## Methods for Studying Transcriptional Enhancers

As mentioned above, active TEs are characterized by low nucleosome density (“openness”), and physical (looping) interactions with their cognate gene promoters, and give rise to small (~500 nt), unstable transcripts known as eRNAs (enhancer RNAs). As such, as well as profiling the chromatin state using ChIP and ChIP-Seq, measurement of these features can be used to identify TEs, both at the level of individual gene loci, and globally. For instance, nucleosome density has commonly been studied using nuclease protection assays, where native chromatin is treated with a nuclease, such as DNAse1, and regulatory regions, including TEs, are identified by their being hypersensitive to digestion because of their low nucleosome density. Indeed, the DNAse 1 hypersensitivity (HS) assay has been adapted to allow genome-wide enhancer mapping (45–47). Recently new and less arduous techniques have also been developed to assay chromatin accessibility, including ATAC-Seq (Assay of Transposon Accessible Chromatin), which measures chromatin openness based on the principle that “closed” regions of the genome are refractory to transposon insertion (48). Therefore, by assaying transposon insertion sites by high-throughput sequencing, openness can be assessed on a genome scale.

**Figure 3:**
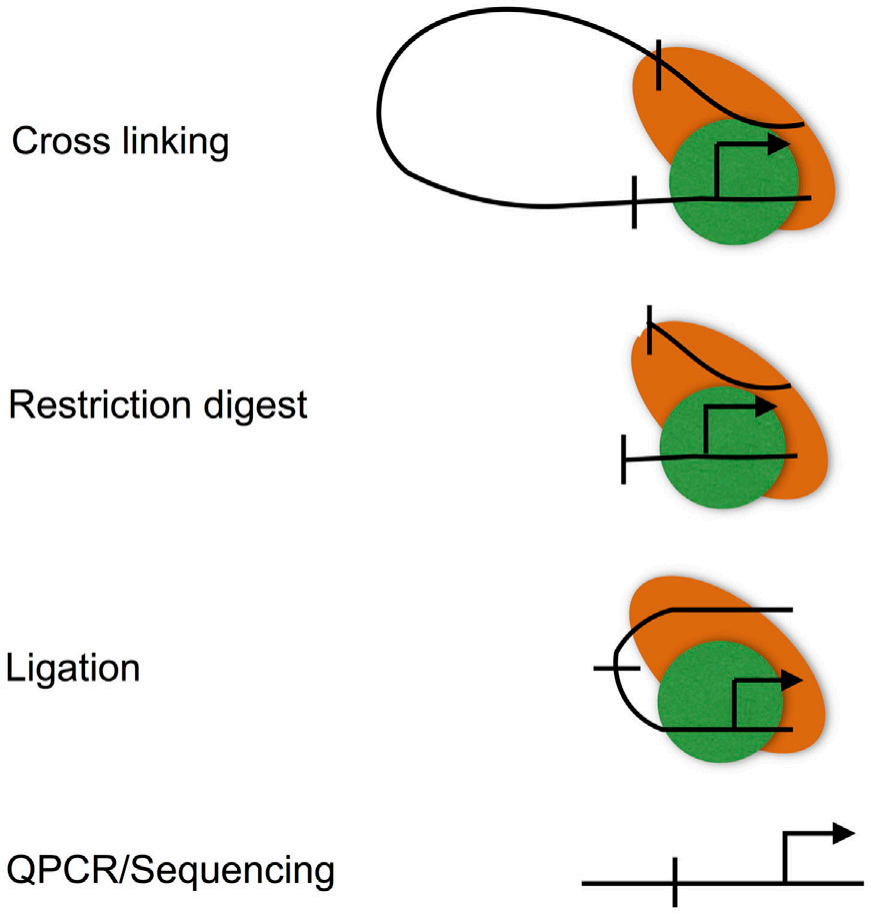
**The chromatin conformation capture (3C) assay**. The 3C assay is used to measure chromatin loops that define promoter–enhancer interactions. Promoter–enhancer interactions are cross-linked, chromatin is then digested with a restriction enzyme, and the resulting restriction fragments are ligated under dilute conditions to favor the ligation of DNA fragments that are in close proximity due to being held together by cross-links. The resulting ligation products are then purified, subjected to quantitative PCR, or high-throughput sequencing (Hi-C).

Chromatin looping is measured using the chromatin conformation capture (3C) assay (Figure 3), and various adaptations to allow locus-wide and genome-wide measurement using high-throughput sequencing technologies (for instance, Hi-C) (49, 50). 3C involves cross-linking the protein–DNA and protein–protein interactions that characterize promoter–enhancer interactions. Cross-linked DNA is then digested with a restriction enzyme, and the resulting restriction fragments are ligated under conditions that favor intra rather than inter molecular ligation, thus allowing enhancers to be ligated to the promoters that they regulate (51). These ligation events can, then, be measured at individual loci by real-time PCR, or genome-wide using high-throughput sequencing.

Finally, CAGE-Seq (Cap Analysis Gene Expression) is used to selectively assay capped RNAs, including mRNAs and eRNAs, by high-throughput sequencing (32, 52). As eRNAs characterize active enhancers, this technique can, therefore, be used for enhancer detection as well as to determine the dynamics of transcription of eRNAs and their cognate gene transcripts.

## Regulation of Type 2 Cytokine Expression by Transcriptional Enhancers

CD4^+^ T cell differentiation has been a particularly useful model for the study of cell fate decisions since the growth factors required to drive naïve CD4^+^ T cells to differentiate multiple effector fates have been identified, allowing differentiation of each fate to be recapitulated *in vitro*. From the point of view of the role of TEs in CD4^+^ T cell lineage commitment, the Th1/Th2 paradigm has been particularly useful. Briefly, the Th1 and Th2 differentiation pathways represent alternate and opposing fate decisions since engagement of one differentiation program represses the other. For instance, the Th2 lineage is defined by expression of the cytokine IL-4, and differentiation of this lineage is dependent on the TF GATA3, which directly induces Th2 genes, including *IL-4*, while repressing Th1 genes, such as *Ifng* (encoding interferon-γ) and *Tbx21* (encoding TBET), both of which drive Th1 development. Thus, GATA3 both induces Th2 differentiation, while enforcing repression of the Th1 program [reviewed in Ref. (1, 9)].

**Figure 4:**
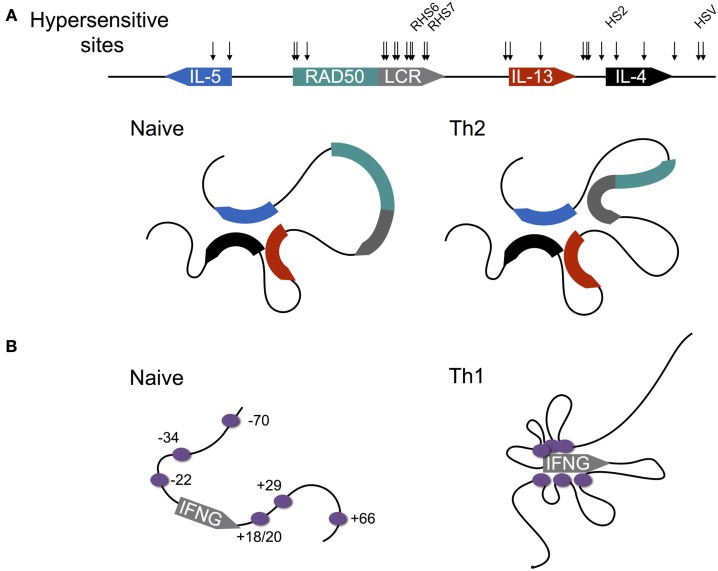
**Differentiation-dependent chromatin looping of the type 2 and interferon gamma loci**. (**A)** In naïve CD4^+^ T cells, the promoters of the genes encoding IL-4, IL-5, and IL-13 are clustered together. Following Th2 differentiation, the locus control region (LCR) contained within a 3′ intron of the *Rad50* gene loops onto the clustered promoters, licensing cytokine expression. DNAse1 hypersensitive sites are shown by arrows, with sites shown to be critical for cytokine expression named. **(B)** In contrast to the type 2 locus, chromatin loops at the murine interferon-gamma (IFNG) locus are acquired as naïve CD4^+^ T cells differentiate to the Th1 fate. Enhancers are shown by purple circles with their coordinates shown relative to the transcription start site (0 kb).

IL-4 is encoded within the type 2 locus that also encodes the Th2 cytokines IL-5 and IL-13 (Figure 4A). Expression of these three genes is co-regulated via a mechanism that involves chromatin looping such that the three gene promoters are in physical interaction (53–56). Interestingly, this interaction is preconfigured in naïve CD4^+^ T cells, despite the fact that naïve T cells are not immediately competent for transcription of the type 2 gene cluster. Indeed, this arrangement is also present in B cells and fibroblasts, both of which are capable of expressing IL-4 and IL-13, and thus the looping mechanisms that regulate transcription of the type 2 cluster in CD4^+^ T cells are not cell type-specific. Within T cells, the preconfigured state of the type 2 locus is critically dependent on the STAT6 TF, since in Stat6^−/−^ cells, chromatin looping is dramatically reduced (55). The reason for this dependence appears to be that STAT6 directly binds throughout the type 2 locus, and therefore may be involved in mediating looping interactions. Moreover, STAT6 is also required for up-regulation of GATA3, which is itself required for type 2 locus expression (53).

In order to gain transcriptional competence, the locus control region (LCR) located within an intron of the *Rad50* gene, which separates *IL-5* from the other genes of the cluster, has to loop onto the clustered gene promoters of the type 2 genes, presumably in order to deliver a factor that licenses transcription. Recruitment of the LCR to the type 2 promoter cluster is likely dependent on GATA3, since overexpression of GATA3 within fibroblasts, which have a preconfigured promoter cluster, but cannot recruit the LCR to activate transcription following stimulation, enables LCR recruitment (55). Indeed GATA3 binds the LCR, suggesting that GATA3 is involved in mediating looping (53, 55, 56).

Early studies of the type 2 locus found at least 15 regions of DNAse1 HS spread throughout the locus in Th2 cells, suggesting that regulatory circuits controlling expression of the type 2 cytokines were either highly complex, functionally redundant, or a combination of both (57–59). The groups of Flavell, Kubo, and Ansel have addressed this question directly, through the generation of mice with deletions of different hypersensitive sites. To date, three regions have been shown to be essential for wild-type (WT) expression of the type 2 locus in Th2 cells and one region within T follicular helper cells because they contain elements that positively control gene expression; Tanaka et al. showed that a site within the second intron of *IL-4* (HS2) is critical for IL-4 expression since its deletion largely abrogated IL-4 production by *in vitro* cultured Th2 cells, and largely ablated the (IL-4-dependent) asthmatic response of HS2-deficient mice *in vivo* (56). Moreover, these defects were likely due to the deletion of a GATA3 binding site within HS2, because unlike WT cells, HS2-deficient CD4^+^ T cells did not produce IL-4 upon GATA3 overexpression. Finally, HS2 deletion resulted in reduced levels of histone acetylation and H3K4 methylation at various positions across the type 2 locus in Th2 cells, indicating that GATA3 binding at HS2 is required to establish a transcriptionally permissive chromatin landscape. Providing a mechanism for GATA3 dependent chromatin remodeling at the type 2 locus, Hosokawa et al. showed that GATA3 is required for recruitment of the histone acetyltransferase p300 to several positions within the type 2 locus, including HS2, the LCR, and all three cytokine promoters (60). The same work showed that within Th2 cells, GATA3 targets a HDAC to a TE of *Tbx21* (encoding TBET), resulting in gene repression. Thus, the same TF associates with both positive and negative regulators of chromatin accessibility within the same cell lineage, enabling it to both drive Th2 differentiation, and to repress Th1 differentiation.

Richard Flavell’s group showed that as per HS2, deletion of hypersensitive sites RHS6 and RHS7, which occur within the type 2 LCR and also serve as sites for GATA3 binding, largely abolished IL-4 production, although it is notable that these deletions also diminished IL-5 and IL-13 expression, which were unaffected following HS2 deletion (56). Moreover, as with HS2, deletion of RHS6 resulted in reduced deposition of histone acetylation and H3K4me3 at the IL-4, IL-5, and IL-13 promoters under type 2 conditions, while an increased deposition of the repressive H3K27me3 modification was observed, suggesting that GATA3 binding is required at several regions of the type 2 locus to allow establishment of a permissive chromatin signature. The effect of RHS7 deletion appears to be through changes to the chromatin looping behavior of the type 2 locus since deletion of RHS7 blocks LCR recruitment to the promoter cluster, indicating that recruitment of the LCR may deliver factors that remodel the type 2 cytokine promoters to facilitate transcription (54). The group of Kohwi-Shigematsu demonstrated using RNA interference that looping interactions at the type 2 locus are largely mediated by the genome organizing protein, SATB1, which binds the type 2 locus at multiple positions, including within RHS7 (53). Thus, taken together, these data indicate that RHS7 positively regulates expression of the type 2 cytokines through binding of SATB1, which in turn, facilitates the necessary chromatin looping interactions. Finally, Vijayanand et al. showed that while deletion of HS V – a HS site occurring downstream of *IL-4* – had little or no effect on type 2 cytokine expression in Th2 cells, expression of both IL-4 and IL-13 was reduced in follicular helper T cells (61). Thus it appears that a combination of the complexity of the regulatory mechanism, and the need to specify transcription in multiple cell lineages may explain the number of type 2 locus regulatory regions.

## Transcriptional Enhancers Regulate Differentiation-Dependent Interferon-γ Expression

In contrast to the type 2 locus, where chromatin loops are largely preconfigured, the interferon gamma (*Ifng*) locus lacks any demonstrated enhancer looping within naïve CD4^+^ T cells (Figure 4B) (62). However, regulation of IFNG expression is similarly complex, with 12 TEs identified to date, most of which contain binding sites for the Th1 defining TF T-BET (encoded by *Tbx21*) [reviewed by Balasubramani et al. (63)], which is required for IFNG expression (64). Indeed Sekimata et al. demonstrated that the formation of chromatin loops within Th1 cells is dependent on T-BET, since in T-BET deficient cells, most of the looping interactions that characterize WT Th1 cells are greatly diminished (62). Moreover, they provided a likely mechanism: T-BET deficiency reduced binding of the insulator factor CTCF at the *Ifng* promoter and + 66 kb enhancer. In turn, CTCF is required for looping at the *Ifng* locus, since knock-down of CTCF diminished looping, but importantly, did not reduce T-BET binding. Thus, taken together, these data indicated that T-BET likely recruits CTCF to the *Ifng* locus, enabling the chromatin looping interactions that facilitate IFNG expression.

The question of the importance of one of the *Ifng* TE (−22 kb) has been assessed by the generation of a knock-out mouse, the result being significantly reduced expression of IFNG in Th1 cells as well as in effector CD8^+^ T cells and natural killer cells (65). Moreover, deletion correlated with changes in the histone modification landscape of the *Ifng* locus, with reduction in permissive H3K4 methylation and H3K12 acetylation modifications in Th1 cells, relative to the WT. Recruitment of RNA polymerase to the *Ifng* gene promoter was also reduced in −22 deficient Th1 cells, indicating that the deletion alters gene expression by directly impacting the initiation of gene transcription, fitting with the notion that enhancer looping licenses gene transcription.

The question of the functional redundancy of the *Ifng* regulatory regions has also been addressed through transgene studies, whereby transgenic mice carrying fragments of the human *Ifng* locus were generated. Using this approach, Thomas Aune’s group have defined four regulatory regions that are essential for WT IFNG expression in human cells: an element occurring at −30 kb (corresponding to the −34 kb enhancer of mice), which is required for WT expression in Th1 cells, effector CD8^+^ T cells and Natural Killer T cells (NKT cells), but not in NK cells; a +20 kb enhancer, which is required for IFNG expression by memory Th1 and NKT cells, and a −4 kb enhancer, which is required for Th1 IFNG expression (66, 67). Finally, they defined a −16 kb region that harbors repressive function since deletion resulted in increased expression of IFNG within Th1, Th2, and effector CD8^+^ T cells. Thus, taken together with the data described above, these studies of the *Ifng* locus support the notion that multiple enhancers are required to specify transcription within different cell types and between differentiation states.

### Regulation of CD8α expression during T cell development and effector differentiation

A well defined example of TE mediated regulation within CD8^+^ T cells is provided by the regulation of CD8α itself. Indeed, as CD8α is expressed by cell types other than CD8^+^ T cells, including CD8^+^ dendritic cells and some intestinal intraepithelial lymphocytes (IELs), studies of CD8α expression have been particularly informative in terms of the role of TEs in cell type-specific regulation. Within conventional T cells, CD8α is up-regulated at the double positive (CD4^+^CD8^+^; DP) stage of thymic development, and subsequently repressed or permanently expressed at the expense of CD4 as T cells commit to either the CD4 or CD8 T cell lineage, respectively. Expression of CD8α is controlled by five TEs (E8_I–V_), which occur upstream of *Cd8*α with E8_IV_ occurring within an intron of *Cd8*β [reviewed in Ref. (68)]. The groups of Littman and Ellmeier have assessed the role of each of the *Cd8*α enhancers through the generation of knock-out mice and the use of transgene reporter assays. For instance, while deletion of E8_I_ has no effect on the development of conventional αβ CD8^+^ T cells, it largely blocks the development of αα CD8^+^ IEL which develops extrathymically (69). Therefore, it appears that different environmental signals engage different *Cd8*α enhancers to establish CD8α expression. Interestingly however, E8_I_ is required for stable CD8α expression following effector differentiation of conventional αβ CD8^+^ T cells (70). These data were surprising in light of earlier findings that E8_I_ drives transgene expression in DP, CD8 SP, and mature peripheral CD8^+^ T cells, and suggested that another element could compensate for the deletion of E8_I_. Indeed, Ellmeier et al. had previously shown that E8_II_ compensated for loss of E8_I_ by generating mice that lacked both enhancer elements, since the mice displayed variegated expression of CD8α at the DP stage (71). Taken together, studies of the *Cd8*α locus illustrate that multiple TEs are required to allow cell type and differentiation state specific transcription, however, unlike the *Ifng* and type 2 loci, some degree of functional redundancy also exists.

Providing an explanation for the functional requirement of E8_I_ and E8_II_ described above, Billic et al. showed that deletion of DNA Methyltransferase 1 (DNMT1) in E8_I_ E8_II_ double-deficient mice partially restored CD8α expression, indicating that E8_I_ and E8_II_ function in recruiting factors that result in removal of CpG methylation (72). Further, while WT DP cells have low levels of CpG methylation at a number of regions within E8_v_, these regions are hypermethylated in E8_I_ and E8_II_ (double) deletion mice, further arguing that E8_I_ and E8_II_ function to recruit factors that demethylate the *Cd8*α locus. In agreement with this, Harland et al. have shown that the CpG methylation status of E8_v_ as well as the *Cd8*α promoter and two intergenic regions is inversely correlated with CD8α expression by assaying methylation from the DN stage, through DP, SP and mature CD8^+^ T cells stages (73). The latter study also showed that when effector CD8^+^ T cells were differentiated in the presence of IL-4, CD8α expression was down-regulated, which coincided with increased CpG methylation at the regions they assayed. Thus it appears that modulated deposition of methyl CpG at multiple sites of the *Cd8*α locus, including the promoter and E_V_, regulates CD8α expression. Moreover, E_I_ and E_II_ are likely to be required for the recruitment of factors responsible for demethylation of the locus, which is a perquisite for CD8α expression.

Finally, the group of Kioussis have shown that CD8α expression correlates positively with looping of enhancers I–IV of the CD8α locus onto the CD8α gene promoter (E8_V_ was not studied) (74). Moreover, using 3D Fluorescence *In situ* Hybridization (FISH), they showed that the positioning of the CD8α locus within the nucleus is also modulated, with expression coinciding with repositioning of the locus, presumably toward regions of the nucleus that are dense with RNA polymerase (“transcription factories”). Thus, it would be interesting to determine whether deletion of specific enhancers, as described above, results in aberrant CD8α expression because of a failure to properly position the locus within CD8^+^ T cells.

## Insights into T Cell Enhancer Function from Whole Genome Studies

The advent of high-throughput sequencing-based technologies has allowed the generality of observations made at individual gene loci to be tested at the level of the genome. In turn, these approaches have provided novel insights into the processes regulating cellular differentiation. For example, Vahedi et al. recently asked how the STAT family of TFs regulates Th1/Th2 differentiation by comparing enhancer usage in the two cell types within WT and STAT deficient mice (44). STAT TFs are key regulators of T cell differentiation, providing a direct link between signaling events occurring at the cell membrane which initiate cellular differentiation, and the transcriptional changes that result. In particular, STAT proteins enable integration of cytokine mediated signaling to shape appropriate T cell differentiation. For instance, IL-4 signals received via the IL-4 receptor result in STAT6 phosphorylation, homodimerisation, and translocation to the nucleus. Once in the nucleus, STAT6 directly binds to the genome, regulating transcription of genes, such as GATA3, which in turn drive Th2 differentiation. Other examples include IL-2 signaling via STAT5, which drives Treg differentiation, and IFN-γ signaling via STAT1, which drives Th1 differentiation [reviewed in Ref. (75)].

While the importance of STAT binding to gene promoters has been clear for some time, binding of STATs to enhancer regions has recently become recognized as equally essential for T cell lineage specification and function. Vahedi et al. showed that while many enhancers (defined as p300^+^ H3K4me1^+^) were shared by Th1 and Th2 cells (~13,000), each lineage had a surprisingly large number of unique enhancers (~9,000 for Th1 cells and ~7,000 for Th2 cells), given the close relationship between the cell types. The authors further demonstrated that many of the differences in the distribution of p300, a marker of lineage-specific enhancers, between Th1 and Th2 were explained by the action of STAT family TFs: by comparing p300 binding in WT and STAT6^−/−^ mice, they showed that ~5,500 p300 sites disappeared from Th2 cells in the absence of STAT6, while the enrichment of H3K4me1 at those sites was also reduced. Furthermore, STAT4^−/−^ mice had over 3,000 p300 peaks absent in Th1 cells relative to the WT. Indeed STAT4 binding accounted for nearly double the number of p300 peaks accounted for by T-BET – the master regulator of Th1 differentiation. Thus, this study highlighted a key role for STAT family TFs in establishing cell type specific enhancer usage, and in turn, the establishment of the transcriptional programs that define Th1 and Th2 cells.

The finding (described earlier) that type 2 effector cytokines are co-regulated via shared promoter and enhancer interactions raises questions about the generality of that mechanism. Indeed, since other immune genes, including those encoding chemokines and chemokine receptor also occur as closely spaced, conserved gene clusters (76), it is possible that the mechanism described for the type 2 locus might be used to co-regulate other immune genes. Keji Zhao’s group recently used a technique called chromatin interaction analysis by paired-end tag sequencing (ChIA-PET), which combines ChIP with 3C to detect looping events that are associated with the binding of particular proteins, to detect similar interactions in human CD4^+^ T cells (77). In their study, Zhao’s group used H3K4me2 as the target for ChIP to enrich interactions involving active gene enhancers and promoters. They found that transcription of lineage-specific genes is controlled by networks of enhancer–promoter and promoter–promoter interactions. Specifically, they found that single enhancers could interact with multiple promoters that a large number of promoters interact with multiple enhancers, and surprisingly, ~3,600 instances of promoter–promoter interactions. Importantly, they found that genes with promoters that have interacting enhancers were more highly transcribed, and the level of transcription scaled with the number of enhancers interacting with the promoter. Finally, genes linked by promoter–promoter interactions were co-transcribed in a tissue-specific manner, indicating that these interactions may achieve the coordination of functionally related genes. Moreover, these observations were in broad agreement with Li et al., who performed ChIA-PET on four varied human cell lines using an antibody against RNAPII, and thus are likely to represent a general means by which lineage-specific transcriptional programs are coordinated (78).

Finally, the studies described above have probed the question of the roles and mechanisms of TE-mediated gene regulation during normal T cell development; however, it has recently become apparent that TEs also have roles to play in disease states. Seumois et al. demonstrated that genome-wide enhancer profiling may be used to delineate the mechanistic basis of autoimmune disease (79). In this study, the authors performed ChIP-Seq for a single histone modification (H3K4me2) that marks poised and active TEs, comparing asthmatic and healthy patients. They found that differences in enhancer usage and the extent of H3K4me2 enrichment existed within the naïve, Th1 and Th2 CD4^+^ T cells of healthy versus asthmatic patients, but tellingly, the differences were greatest within memory Th2 phenotype cells, which is of interest because aberrant accumulation of memory Th2 cells is an important step in asthma development. Supporting the notion that these changes are causative, the asthma-associated enhancers discovered by this study mapped to a number of regions that regulate asthma associated genes. For instance, three regions mapping to the LCR of the type 2 locus showed increased enrichment for H3K4me2 in asthma derived Th2 cells, relative to the healthy controls. Finally, as far as a mechanism by which differential enhancer usage may impact disease progression, they found that ~40% of the asthma-associated enhancers contained binding sites for TFs that are associated with T cell differentiation, including GATA3 and TBET. Thus, it appears that differences in enhancer usage impacts expression of genes associated with asthma progression by influencing TF binding, although the reason that the enhancer profiles of healthy and asthmatic patients differed to begin with is yet to be determined. Consistent with the observations of Seumois et al., John O’Shea’s group has shown that mapping of so-called super-enhancers (SEs) within differentiating T cells enabled the delineation of disease-associated regulatory networks (80). SEs are defined as clusters of conventional TEs that have close spacing, which, when active, are enriched for lineage specifying TFs, and the transcriptional co-activators p300 and Mediator of RNA polymerase II subunit 1 (Med1) [reviewed in Ref. (81)]. Importantly, SEs are highly enriched at lineage defining genes. For instance, studying Th1, Th2 and Th17 cells, the O’Shea study showed that SEs characterized cytokine and cytokine receptor encoding loci, as expected for helper T cells, and were enriched for TFs that defined each lineage – T-BET within Th1 cells, GATA3 in Th2 cells, and ROR-γt in Th17 cells. The SEs were also highly enriched for STAT family proteins, consistent with the finding that STAT proteins are essential for the establishment of the lineage-specific “enhancersomes” of Th1 and Th2 cells. Interestingly, a SE at the BACH2 locus was active in all three lineages, and BACH2 binding sites were highly enriched within the SEs of each cell type. By performing RNA-Seq on BACH2 proficient and sufficient mice, they could show that BACH2 represses effector functions associated with each T cell lineage. Finally, they showed that SNPs associated with autoimmune diseases including rheumatoid arthritis and type 1 diabetes, but not SNPs associated with non-autoimmune diseases, such as cancer or type 2 diabetes, were enriched within SEs relative to conventional enhancers. Thus, consistent with the findings of Seumois et al., lineage specifying TEs appear to be hotspots for potentially pathogenic SNPs.

## Concluding Remarks

Transcriptional enhancers have key roles to play in the differentiation of T cells, as well as in the development of disease states, including autoimmune diseases, indicating that a thorough understanding of their biology may lead to opportunities to modulate immunity for benefit as well as to understand the mechanistic basis of immune pathologies. Much of our knowledge of the role of TEs as regulators of T cell differentiation has come from the generation of mice baring specific enhancer deletions. For instance, this strategy has been critical to our understanding of the regulation of the type 2 locus, which serves as one of the most detailed paradigms of enhancer-mediated gene regulation in any cell type. With recent advances in our ability to rapidly generate mice with precise genetic lesions, including the CRISPR/Cas9 technology [reviewed in Ref. (82)], these types of studies should progress at an increasing rate. Moreover, as technological advances are starting to enable transcriptional regulation to be probed within single cells, many questions that were previously impossible to address are becoming tractable. In particular, studies of rare populations such as naïve precursors and antigen-specific memory T cells can now begin to be asked. These include questions about the role that TEs play in the establishment and maintenance of immune memory, and memory recall capacity, and whether or not differential enhancer engagement might explain differences between highly efficacious vaccines, and those that confer weak or waning immunity.

## Conflict of Interest Statement

The authors declare that the research was conducted in the absence of any commercial or financial relationships that could be construed as a potential conflict of interest.
